# Complementary feeding methods and introduction of ultra-processed foods: A randomized clinical trial

**DOI:** 10.3389/fnut.2022.1043400

**Published:** 2022-12-07

**Authors:** Paula Ruffoni Moreira, Leandro Meirelles Nunes, Elsa Regina Justo Giugliani, Erissandra Gomes, Jordana Führ, Renata Oliveira Neves, Christy Hannah Sanini Belin, Juliana Rombaldi Bernardi

**Affiliations:** ^1^Hospital de Clínicas de Porto Alegre (HCPA), Porto Alegre, Brazil; ^2^Graduate Program in Food, Nutrition, and Health, Department of Nutrition, School of Medicine, Universidade Federal do Rio Grande do Sul (UFRGS), Porto Alegre, Brazil; ^3^Graduate Program in Child and Adolescent Health, Department of Pediatrics, School of Medicine, Universidade Federal do Rio Grande do Sul (UFRGS), Porto Alegre, Brazil; ^4^School of Dentistry, Department of Surgery and Orthopedics, Universidade Federal do Rio Grande do Sul (UFRGS), Porto Alegre, Brazil

**Keywords:** complementary feeding, nutrition, complementary foods, nutritional interventions, child nutrition, randomized clinical trial

## Abstract

**Introduction:**

Complementary feeding (CF) is defined as a period when foods, other than milk, are introduced to the infant’s diet. Unfortunately, frequent consumption of ultra-processed foods (UPF) has become highly prevalent early in an infant’s life. The aim was to verify the association of CF methods with the introduction of UPF in early childhood.

**Methods:**

This randomized clinical trial involved pairs of mother-infants, allocated in groups receiving different CF interventions: strict Parent-Led Weaning (PLW); strict Baby-Led Introduction to SolidS (BLISS), or mixed-method. The intervention consisted of a counseling session on healthy eating at the child’s 5.5 months of age. A structured questionnaire was created based on the NOVA classification for the definition of UPF and applied at 9 and 12 months. The effect of the CF method intervention was measured by a survival curve for UPF offered for the first time in early childhood between groups. Cox regression was used to estimate its magnitude. The primary analysis was done in three groups (PLW, BLISS, and Mixed) and the secondary analysis was done in two groups (PLW, and BLISS + Mixed).

**Results:**

A total of 139 mother-infant pairs were eligible and 129 followed the study. The prevalence of infants who were exposed to UPF in early childhood was 58.9% (*n* = 76), being 71.4% in the PLW group, 53.3% in the BLISS group, and 52.4% in the Mixed group, without differences between them (*p* = 0.133). The PLW group intervention had a greater chance of exposure to ice cream or popsicles (*p* = 0.032) and sweet crackers (*p* = 0.009), compared with the other two CF groups. The Cox regression did not find significant differences between the three groups. However, the regression with two groups estimated a 38% reduction in the offer of UPF in the BLISS + Mixed group intervention (*p* = 0.049).

**Discussion:**

The CF intervention promoting greater infant autonomy (BLISS and Mixed) was associated with a reduction in the offer of UPF in early childhood. This knowledge may contribute to supporting strategies aimed at reducing UPF consumption by the young infant.

**Brazilian registry of clinical trials (ReBEC):**

[https://ensaiosclinicos.gov.br/rg/RBR-229scm], identifier [RBR-229scm U1111-1226-9516].

## Introduction

According to the World Health Organization (WHO), complementary feeding (CF) is recommended when breast milk is no longer sufficient to meet the nutritional requirements of infants, and therefore other foods and liquids are needed, along with breast milk (1). The process, generally between 6 and 23 months of age, represents the transition from milk feeding to family foods (2, 3).

Usually, at the beginning of CF, children receive mashed foods offered with a spoon by an adult (4). This method of feeding is also called Parent-Led Weaning (PLW) and is majority guided by the adult that is offering the food. However, in the last decades, new methods of CF have been proposed, such as Baby-led Weaning (BLW) and Baby-led Introduction to SolidS (BLISS). Both advocate the introduction of unprocessed and minimally processed foods in a way that infants can put the food in their mouths by themselves (5, 6). These infant-guided methods seem to be beneficial by reducing infant food fussiness, increasing satiety responsiveness, and encouraging infants to improve their oral motor skills (7).

The ultra-processed foods (UPFs) are industrial formulations that typically include substances not commonly used in culinary preparations, and additives whose purpose is to imitate the sensory qualities of unprocessed foods (8), which included: soft drinks; packaged snacks and candies; mass-produced packaged bread and buns, cookies, pastries, cakes; margarine and other spreads; breakfast cereals; pre-prepared meat, cheese, pasta, and pizza dishes; poultry and fish nuggets and sticks; sausages, burgers, hot dogs, among other foods marketed (9).

The offer of UPFs is present in the diet of 43.1–90.6% of children under 24 months of age in Brazil (10, 11), and 53.7–91.2% in other populations (12). The most consumed UPFs among Brazilian children are artificial juice (nectar, concentrated drink, or refreshment), yogurt/dairy drink, soda, Petit-Suisse, crackers/biscuits, instant noodles, sweets (candies), and chocolate milk (13). Recent literature reviews confirmed that UPF consumption is associated with poor dietary quality and with adverse metabolic and health outcomes throughout life (14, 15). Longitudinal studies about its consumption at preschool age found a significant association with a higher increase in total cholesterol and Low-Density Lipoprotein (LDL) cholesterol (16), a significant increase in waist circumference from preschool to school age (17), and greater increases in adiposity from childhood to early adulthood (18).

Despite the increasing popularity and adherence to new methods of CF, there are few studies evaluating the impact of these methods on the introduction of unhealthy foods or UPFs (19, 20). The available data suggest that children feeding by BLW and BLISS methods have lower use of salt and sugar added, common ingredients in UPF (21). Given this scenario of the high consumption of ultra-processed foods in young children, strategies are needed to reduce this consumption. For this, it is necessary to know practices and behaviors associated with greater or lesser consumption of these foods.

In this context, this study aimed to verify whether interventions on different methods of complementary foods are associated with the introduction of UPFs in the diet of young children.

## Materials and methods

### Study design

This is a randomized clinical trial comparing three different groups of infants regarding the method of food introduction: strict Parent-Led Weaning (PLW): an approach conducted by the caregiver in which children are mostly spoon-fed; strict BLISS: a technique guided by the child, in which they feed themselves–there are no spoon-feeding or purees; mixed-method (Mixed): a combination of PLW and BLISS, according to the child’s wishes for each food preparation, i.e., parents were instructed to initially apply the BLISS approach. If the child was not satisfied or showed disinterest, they were instructed to offer the food using the PLW technique during the same meal. The randomized clinical trial was designed to identify differences in health outcomes between groups (22, 23).

### Participants

The sample was recruited by an online invitation, through social networking pages, targeted to mothers’ groups, through newspaper ads, and on a Southern Brazil hospital bulletin board, between the years 2019 and 2020. An email address and a phone number were provided for interested mothers to make the first contact with the researchers showing interest in participating. At this moment, a standardized text explaining the intervention, household visits, and the need to commute to the hospital at 12 months of the children’s age was given, in addition to verifying whether the child met the inclusion criteria (healthy singleton infants with birth weight greater than >2.500 grams and gestational age ≥37 weeks, internet access, living in Porto Alegre, RS, Brazil, or nearby cities and should not have started CF yet).

After checking the inclusion criteria, the mothers signed the free and informed consent form online. Behind signing, the participants were sequentially numbered and had their identification numbers entered into a randomization list of three blocks and equal numbers, previously computer-generated^1^ by a blinded researcher, that did not have contact with the participants during the recruitment or the data collection. Participants were enrolled and assigned by different study group researchers.

### Intervention

The detailed intervention, performed at 5.5 months of children’s age, was published previously (22, 23). Briefly, it consisted of a dietary workshop, carry out at a private nutrition office equipped with a test kitchen, in which a nutritionist cooked in real-time examples of baby food and explained standardized information about the CF method to the participants, that were blind to the allocation group until the intervention day. The nutritionist was previously informed about what method she would teach, and the blindness was guaranteed with a different researcher contacting the participants. Regardless of the allocated group, the dietary workshop promoted healthy eating, based on the “Dietary Guidelines for Brazilian Children Under Two Years of Age,” by the Ministry of Health of Brazil (4). It consists in offering mostly unprocessed or minimally processed foods, with a minority offer of culinary ingredients and processed foods; being the offer of UPF discouraged.

Parents were encouraged to offer fruits as snacks during the first year of life and stimulated to postpone the use of ready-to-eat meals. Freezing techniques were also taught as an alternative to reduce the preparation time of dinner and lunch meals. At the end of the intervention, an illustrated pamphlet was given summarizing the information and listing examples of UPF that should not offer before 2 years of age. The nutritionist’s phone number and email address were available to the family during the first 12 months of the child, to provide any extra support needed or to report adverse events.

### Data collection

Sociodemographic (maternal age, family income, maternal education, marital status, parity, and child’s sex) variables were collected through a questionnaire sent online to the mothers after signing the free and informed consent form.

In two moments, at nine and 12 months of age, a structured questionnaire about the offer of UPF was applied to ask if the mother had ever offered any UPF from a list and, if positive, how old the child was at the moment of this first exposure (Supplementary material). Likewise, the parents answered questions about exclusive breastfeeding (EBF), any breastfeeding (BF), and CF introduction.

Because of the COVID-19 pandemic, initiated in March 2020, presential collections were suspended, and questionnaires were answered online at 9 months by 50.7% (*n* = 67) mothers, and after 12 months by 80.3% (*n* = 94), between March 2020 and March 2021.

Exclusive breastfeeding practice was defined as when the child received no liquid or solid other than human milk–not even water–except the oral rehydration solution, or drops/syrups of vitamins, minerals, or medications. Any BF practice was defined as receiving any amount of human milk by bottle, cup, or breast, independent of any other food offering (24).

Foods were categorized according to the degree of food processing using the NOVA classification (9, 25, 26), which defines UPF as products with multiple ingredients and stages of processing techniques, many of them exclusively for industrial use. The authors listed the most frequently consumed products during childhood. This list was created based on the most popular consumed products in this period of life according to the “Dietary Guidelines for Brazilian Children Under Two Years of Age” (4), which comprehended: chocolate milk, soft drinks, industrialized baby food, processed meat, sandwich cookies, sweet crackers, salty snacks, chocolate, candies, gelatin, ice cream or popsicle, and artificial juice.

### Sample size

The sample size was calculated by the online version of Power and Sample Size for Health Researchers (PSS Health^®^, Porto Alegre, Brazil) to detect a difference in the exposure to UPF offer of 30% (27). For a power of 80% at a significance level of 5%, based on two-sided testing, including 5% of patients lost to follow-up, the estimated minimum sample size was 132 patients (42 per group).

### Statistical analysis

The database was created using double data entry. Statistical analyses were performed using the software Statistical Package for the Social Sciences^®^ (SPSS, Inc., Chicago, IL, USA) version 22.0 for Windows. The statistical analyses were based on the intention to treat principle. Qualitative variables were expressed by absolute number and percentage, and non-parametric quantitative variables were expressed by the median and interquartile range [P25–P75]. For comparisons, ANOVA one-way test with Tukey’s *post-hoc* was used, as well as the Kolmogorov–Smirnov test to identify the normality of variables.

The survival analysis was used to compare the frequency of initiation of UPF offers in the first 12 months of life between the different groups. The log-rank test was applied to compare the Kaplan–Meier curves, and Cox regression was applied to estimate the magnitude of the association between the intervention and the introduction of UPF in the first year of life, through hazard ratio (HR) and its respective confidence interval (CI) of 95%. The medians of the children’s age at which UPF was introduced for the different groups and respective 95% CI, expressed in days, were also calculated. The statistical significance level adopted was *p* < 0.05.

Initially, the results are presented as a 3-arm trial, as proposed in the protocol of the study; however, to compare the effect of the intervention on UPF offer, the methods that promote greater autonomy (BLISS and mixed) (28) were combined into a single group, because they have similar outcomes in the survival curves and to increase the power of the statistical analysis.

### Ethical aspects

The research was approved by the Research Ethics Committee of the Hospital de Clínicas de Porto Alegre under number 2019-0230 (CAAE: 1537018500005327). The clinical trial was submitted to the Brazilian Registry of Clinical Trials (ReBEC), under number RBR-229scm U1111-1226-9516.

## Results

**FIGURE 1:**
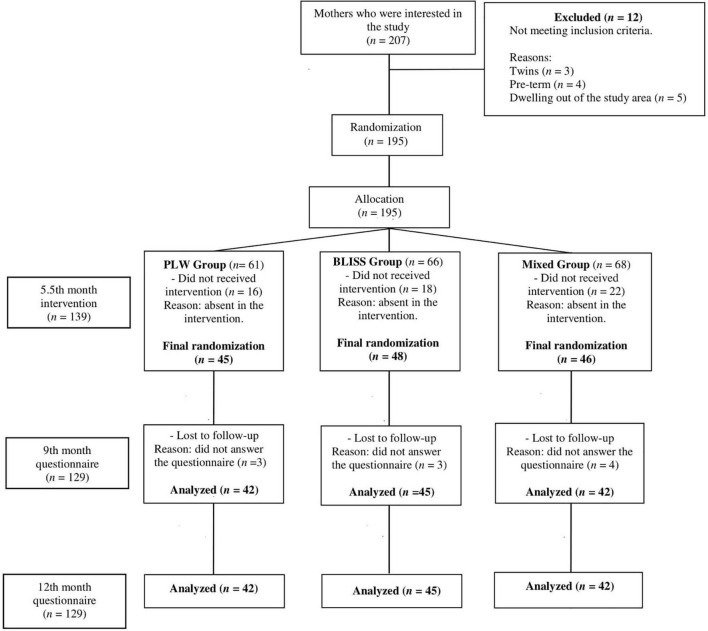
Study design flow chart, Brazil, 2019–2021. PLW, parent-led weaning; BLISS, baby-led introduction to solids.

A total of 207 mother-infant pairs contacted the research team, out of which 12 (5.8%) did not meet the inclusion criteria, leaving 195 mother-infant pairs eligible that were randomized. There were 56 (27.0%) mother-infant pairs who chose not to proceed with the interventions. A total of 139 mother-infant pairs were included in the study, 45 (32.4%) in the PLW group, 48 (34.5%) in the BLISS group, and 46 (33.1%) in the mixed-method group. During the follow-up, 10 mother-infant pairs failed to answer the questionnaires. Finally, data from 129 mother-infant pairs were analyzed in the study. Harms or unintended effects were not reported by participants. The clinical trial profile is shown in Figure 1, from the recruitment of the mother-infants pairs until the evaluation in the 12 month of children’s age.

The characteristics of the mother-infant pairs included in the study are shown in Table 1. There are no statistically significant differences in these variables between intervention groups (*p* ≥ 0.05).

**TABLE 1:** Characteristics of mothers and infants according to interventions groups, Brazil, 2019–2021.

Characteristics	Total	PLW	BLISS	Mixed
*n* (%)	129 (100.0)	42 (32.6)	45 (34.8)	42 (32.6)
**Mothers’ characteristic**				
**Maternal age (years), median [P25-P75]**				
	34 [30–37]	34 [27–37]	35 [32–39]	33 [29–36]
**Parity, *n* (%)**				
Primiparous	106 (82.2)	32 (76.2)	36 (80.0)	38 (90.5)
**Family income (BRL), median [P25-P75]^a^**				
	6.250 [4.000–10.000]	5.000 [3.250–10.000]	8.000 [4.000–14.000]	5.500 [3.875–10.000]
**Maternal education (years), median [P25-P75]**				
	18 [15–20]	16 [13–20]	18 [15–20]	18 [16–20]
**Live with a partner, *n* (%)**				
Yes	110 (85.3)	33 (78.6)	41 (91.1)	36 (85.7)
**Race/ethnicity, *n* (%)**				
White	109 (85.2)	34 (82.9)	38 (84.4)	37 (88.1)
**Infants’ characteristic**				
**Sex, *n* (%)**				
Female	66 (51.2)	24 (57.1)	23 (51.1)	19 (45.2)
**EBF** (up to the 6 months), *n* (%)*				
Yes	78 (62.4)	25 (64.1)	25 (55.6)	28 (68.3)
**Any BF** (at 12 months), ***n* (%)**				
Yes	101 (78.3)	35 (83.3)	35 (77.8)	31 (73.8)

BLISS, baby-led introduction to solids; PLW, parent-led weaning; EBF, exclusive breastfeeding; BF, breastfeeding; P, percentile; *n = 125. Family income expressed in BRL (Brazilian Real)–^a^1 BRL = USD 0.21.

The prevalence of infants who were offered at least once UPF in the first year of life was 58.9% (*n* = 76): PLW group 30/42, 71.4%, BLISS group 24/45, 53.3%, and mixed group 22/42, 52.4%, without statistically significant differences between groups (*p* = 0.133) (data not shown in tables).

The median age of offer to UPF was 300 days [240–365] in the PLW group, 365 days [240–365] in the BLISS group, and 365 days [270–365] in the mixed group. There are no statistically significant differences between the three methods and the age of offer to UPF (*p* = 0.086). Analyzing the PLW group versus the BLISS and Mixed groups together, 300 days [240–365] and 365 days [270–365], respectively, there is a statistically significant difference to offer later in the groups in which the children had greater autonomy (*p* = 0.037) (data not shown in tables).

**FIGURE 2:**
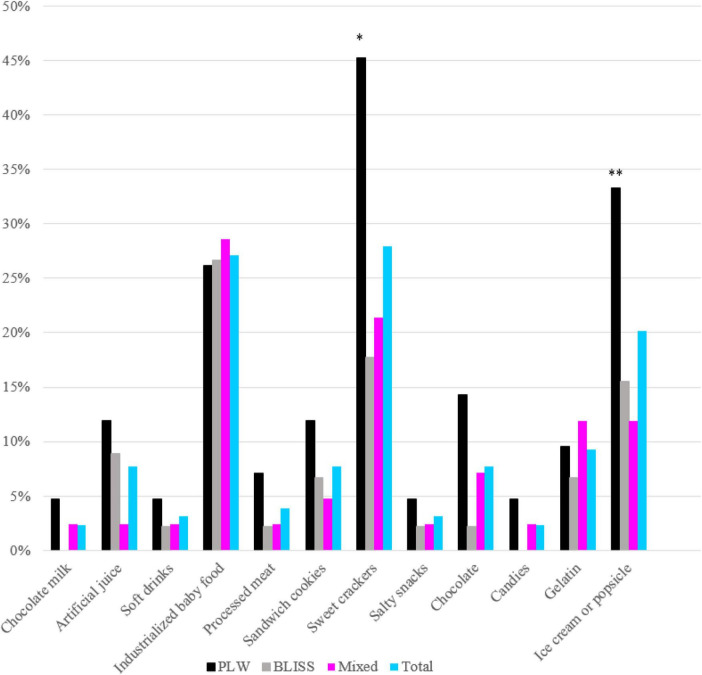
Prevalence of ultra-processed food offered in the first year of life according to interventions: PLW, BLISS, and mixed groups, Brazil, 2019–2021. PLW, parent-led weaning; BLISS, baby-led introduction to solids. *p < 0.05; ^**^p < 0.001.

The offer of each UPF item between groups is shown in Figure 2. The PLW group had a significantly more chance of exposure to children to ice cream or popsicles (*p* = 0.032) and sweet crackers (*p* = 0.009), compared with the other two CF groups.

**FIGURE 3:**
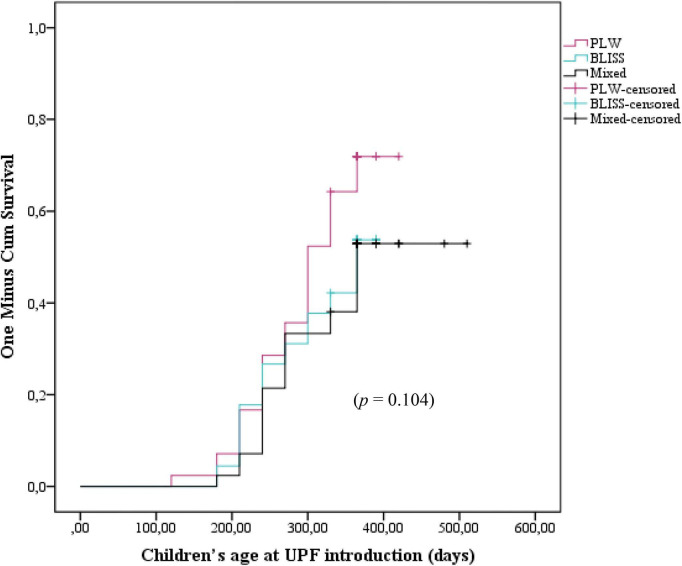
Kaplan–Meier curve displaying the probability of being introduced to ultra-processed foods in the first year of children’s life according to interventions: PLW, BLISS, and mixed groups, Brazil, 2019–2021. PLW, parent-led weaning; BLISS, baby-led introduction to solids.

**FIGURE 4:**
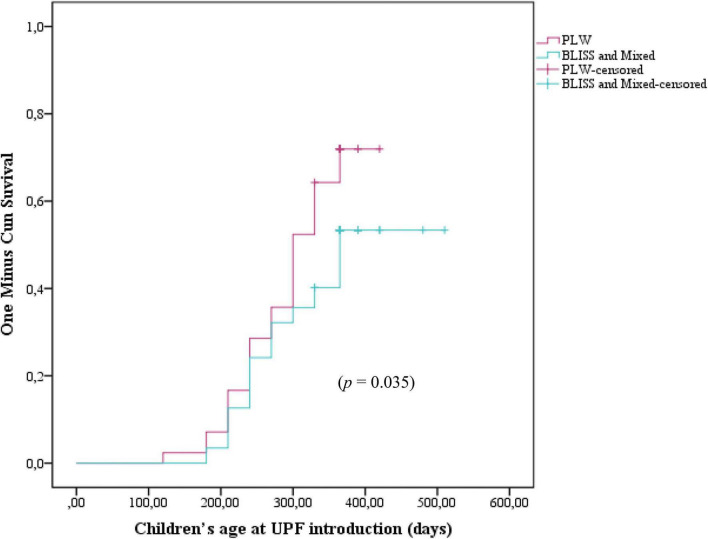
Kaplan–Meier curve displaying the probability of being introduced to ultra-processed foods in the first year of children’s life according to interventions: PLW, BLISS, and mixed groups, Brazil, 2019–2021. PLW, parent-led weaning; BLISS, baby-led introduction to solids.

Figure 3 shows the Kaplan–Meier curves of the initiation of UPF offer to children according to the CF intervention: BLISS, PLW, and Mixed groups. The log-rank test indicated that the curves were not significantly different between the groups (*p* = 0.104). However, by grouping the BLISS and mixed intervention groups the log-rank test indicated that the curves were significantly different between the groups (*p* = 0.035) (Figure 4).

The Cox regression did not find differences between the PLW (control) and the BLISS (HR 1.53; 95% CI 0.89–2.63; *p* = 0.118) and mixed (HR 0.93; 95% CI 0.52–1.66; *p* = 0.808) groups individually. However, by agreeing on the BLISS and mixed intervention methods (two interventions promoting more autonomy for children to eat), the Cox regression estimated a 38% reduction in the UPF offer (HR 0.62; 95% CI 0.39–0.99; *p* = 0.049) (Table 2).

**TABLE 2:** Risk of exposure to ultra-processed foods (UPFs) in the first year of life, according to complementary feeding (CF) interventions groups, Brazil, 2019–2021.

Complementary feeding interventions	HR	CI	*P*-value
PLW	1		
BLISS	1.536	0.897–2.632	0.118
Mixed	0.931	0.522–1.660	0.808
BLISS and mixed	0.629	0.396–0.998	0.049*

PLW, parent-led weaning; BLISS, baby-led introduction to solids; HR, hazard ratio; CI, confidence interval; *p < 0.05.

## Discussion

In this study, children randomized to intervention groups promoting greater autonomy to eat (BLISS and Mixed), were exposed to UPF 65 days after those randomized to the PLW method intervention. Being allocated to BLISS and Mixed groups interventions reduced 38% of the UPF offer in early childhood. The early introduction of UPF, with high-sugar and hyper-palatable foods, can cause taste dysfunctions in early childhood. Children are born with a biological predisposition to prefer sweets, probably an evolutionary adaptation to be attracted to foods rich in energy (carbohydrates) (29). Thus, the posterior exposure among those allocated in BLISS and Mixed methods can be protective concerning the formation of the infant taste.

Even after the intervention on healthy CF and the recommendation not to offer UPF before 24 months, more than half of the children were exposed to UPF in early childhood (58.9%). One study conducted in Brazil demonstrated a prevalence of 31.3% of exposure to UPF in children under 6 months, a period in which the recommendation is for dairy feeding exclusively (30). Another Brazilian study with children under 1 year showed that 87.5% had been exposed to at least one UPF the day before (31). In general, the consumption of UPF is associated with conditions of economic vulnerability (32). However, despite the high income and schooling of the mothers in this sample, we found a high prevalence of exposure to this type of food. In this randomized clinical trial, no differences were found in the income and schooling of randomized mothers for the PLW method intervention that explained the higher and earlier exposure to processed foods.

A recent study in Portugal evidenced that most of the available foods on the market are industrialized and ultra-processed, and the consumption of these foods is greater in the higher-income neighborhood (27). This scenario is similarly found in Brazil (33). According to a recent cross-sectional study, more than 50% of products destined for children under 12 months are classified as UPF in the market, opposite to what Brazilian and international guidelines recommend for this age, making it crucial to implement innovative strategies for parents to improve the CF practices and disseminate correct information regarding food processing (34). A cross-sectional analysis found that 47% of mothers (*n* = 631) did not follow the infants’ healthy eating recommendations received by public health providers. Out of these, 45.7% did not recognize the significance of food on child health even after the professionals’ instructions. The authors of this research state that simply passing information to parents may not be enough to motivate mothers’ actions regarding healthy eating habits (35).

Breastfeeding has been reported to reduce exposure to UPF (36, 37). Children who are breastfed develop a greater acceptance of the flavors present in vegetables, while non-breastfed children have a greater acceptance of sweets (38). So, it is likely that the association between breast milk and UPF is partly due to differences in taste. We found high rates of BF in our sample; however, this did not seem to reduce exposure to UPF. Nevertheless, the effect of BF on UPF intake is not restricted to early childhood. A cohort study showed that BF for more than 4 months of age reduced calorie intake from UPF (39). Although we did not observe a reduction in the supply of UPF in a sample at a rate higher than 60% of EBF, BF should still be encouraged for better taste formation.

Belonging to the intervention groups with greater autonomy delayed the introduction of UPF by 4 weeks in our research. It is likely that the intervention of BLISS and Mixed methods, which promoted the benefits of the child eating whole and fresh foods, aroused in mothers an additional concern not to offer UPF. Additionally, the discouragement of the use of the spoon may have contributed to the lower exposure to ice cream, which may explain the greater exposure to this food in the PLW method. Although the question asked by the mothers did not specify whether the offer was ice cream or popsicle, ice cream was likely the most consuming food among those randomized to the PLW intervention group since this food should preferably be offered per spoon to the child. Another UPF most prevalently offered in the PLW method was sweet crackers, a food that usually children eat by hand. A possible explanation for this is that this type of food is wrongly considered practical to be offered to train children’s autonomy in PLW groups, which is not necessary for other methods.

It is important to note that this study occurred at a time when the new Brazilian infant food guideline was being implemented (40). Previously, the Brazilian infant guideline (4) did not focus heavily on the processing level of the foods offered, as the new guideline does, despite already endorsing healthy food choices. Thus, it is possible that, once the information in the new guideline is implemented and disseminated, the knowledge about UPF will increase and, consequently, their offer can decrease.

This study had limitations and strengths. Since our sample was spontaneously recruited mainly from on-target social networks, it could result in mothers previously interested in healthy eating. The change from in-person questionnaires to online could modify the responses and refer to different sociodemographic characteristics of our population. Furthermore, we did not measure the frequency of exposure of the children to UPF the infant in the first year. As the results were analyzed by the Intention-to-Treat statement, we couldn’t measure adherence to the CF methods. However, it is noteworthy that our results constitute the first known publication exploring the consumption of UPF among three randomized groups submitted to a healthy eating intervention in early childhood, a period crucial to the establishment of healthy habits.

## Conclusion

In conclusion, the infants who were submitted to the interventions using methods of introduction of CF with greater autonomy were less exposed to UPF and were exposed later. In addition, despite the intervention in healthy eating, it is high the prevalence of children exposed to UPF during the first year of life. Further studies are needed to confirm these findings and to explain the association and mechanisms involved with outcomes of child health. This knowledge may contribute to supporting strategies aimed at reducing UPF consumption by young children.

## Data availability statement

The original contributions presented in this study are included in the article/Supplementary material, further inquiries can be directed to the corresponding author.

## Ethics statement

The studies involving human participants were reviewed and approved by Research Ethics Committee of the Hospital de Clínicas de Porto Alegre under number: 2019-0230 (CAAE: 1537018500005327). Written informed consent to participate in this study was provided by the participants’ legal guardian/next of kin.

## Author contributions

LN, JB, and EG conceptualized and designed the study, drafted the initial manuscript, and revised the manuscript. ERJG reviewed the statistical analyses and the manuscript. JF, PM, and CB collected data and carried out the analyses. RN coordinated data collection and statistical analyses. All authors wrote and approved the final manuscript as submitted and agree to be accountable for aspects of the manuscript.

## Footnotes

1
www.randomization.com
