# Supplementary material for: Complementary feeding methods and introduction of ultra-processed foods: A randomized clinical trial
Source: Front Nutr. 2022 Dec 7;9:1043400. doi: 10.3389/fnut.2022.1043400 (PMC9767977; doi:10.3389/fnut.2022.1043400)
Supplement: Supplementary file 1 [file Table_1.doc]

**Supplementary material**

Table S1. The questionnaire sent to mothers on the introduction of food groups

When did you introduce the following foods into your child's meal? (Reply in months)

| Foods | No | 1 | 2 | 3 | 4 | 5 | 6 | 7 | 8 | 9 | 10 | 11 | 12 |
| --- | --- | --- | --- | --- | --- | --- | --- | --- | --- | --- | --- | --- | --- |
| D88) Tubers (yucan, potato, sweet potato, yams) |  |  |  |  |  |  |  |  |  |  |  |  |  |
| D89) Vegetables (cabbage leaves, tomatoes, strawberries, carrots, spinach, beets, broccoli) |  |  |  |  |  |  |  |  |  |  |  |  |  |
| D89) Fruits |  |  |  |  |  |  |  |  |  |  |  |  |  |
| D90) Cereals (rice, pasta, bread, biscuits flour) |  |  |  |  |  |  |  |  |  |  |  |  |  |
| D91) Legumes (beans, lentils, peas, chickpeas, soybeans, broad beans) |  |  |  |  |  |  |  |  |  |  |  |  |  |
| D92) Meat (beef, chicken, pork, fish, viscera) |  |  |  |  |  |  |  |  |  |  |  |  |  |
| D93) Eggs |  |  |  |  |  |  |  |  |  |  |  |  |  |
| D94) Family food |  |  |  |  |  |  |  |  |  |  |  |  |  |
| D95) Milk derivatives (yogurts, Petit-Suisse, cheeses, curd) |  |  |  |  |  |  |  |  |  |  |  |  |  |
| D96) Additional sugar (baby bottle, juice, or tea) |  |  |  |  |  |  |  |  |  |  |  |  |  |
| D97) Honey |  |  |  |  |  |  |  |  |  |  |  |  |  |
| D98) Chocolate milk |  |  |  |  |  |  |  |  |  |  |  |  |  |
| D99) Natural juice |  |  |  |  |  |  |  |  |  |  |  |  |  |
| D100) Industrialized juice (powder or concentrate) |  |  |  |  |  |  |  |  |  |  |  |  |  |
| D101) Soft drink |  |  |  |  |  |  |  |  |  |  |  |  |  |
| D102) Industrialized baby food |  |  |  |  |  |  |  |  |  |  |  |  |  |
| D103) Canned food (tuna, corn) |  |  |  |  |  |  |  |  |  |  |  |  |  |
| D104) Processed meat (presumed, Chester, mortadella, salami...) |  |  |  |  |  |  |  |  |  |  |  |  |  |
| D105) Sandwich cookies |  |  |  |  |  |  |  |  |  |  |  |  |  |
| D106) Sweet crackers |  |  |  |  |  |  |  |  |  |  |  |  |  |
| D107) Salty snacks |  |  |  |  |  |  |  |  |  |  |  |  |  |
| D108) Chocolate |  |  |  |  |  |  |  |  |  |  |  |  |  |
| D109) Candies |  |  |  |  |  |  |  |  |  |  |  |  |  |
| D110) Gelatin |  |  |  |  |  |  |  |  |  |  |  |  |  |
| D111) Ice cream or popsicle |  |  |  |  |  |  |  |  |  |  |  |  |  |
| D112) French fried, fried chicken |  |  |  |  |  |  |  |  |  |  |  |  |  |
